# Civil society engagement in multi-stakeholder dialogue: a qualitative study exploring the opinions and perceptions of MeTA members

**DOI:** 10.1186/s40545-016-0096-0

**Published:** 2017-01-06

**Authors:** Gemma L. Buckland-Merrett, Catherine Kilkenny, Tim Reed

**Affiliations:** Health Action International, Overtoom 60 (2), 1054 HK Amsterdam, The Netherlands

**Keywords:** Multi-stakeholder dialogue, Partnerships, MeTA, Civil society engagement

## Abstract

**Background:**

The Medicines Transparency Alliance (MeTA) is an initiative that brings together all stakeholders in the medicines market to create a multi-stakeholder dialogue and improve access, availability and affordability of medicines. Key to this multi-stakeholder dialogue is the participation of Civil Society Organisations. A recent MeTA annual review, identified uneven engagement of civil society organisations in the multi-stakeholder process. This study was designed to explore the engagement of Civil Society Organisations in the MeTA multi-stakeholder process and the factors influencing their participation.

**Methods:**

Participants were drawn from a convenience sample of key MeTA informants attending a MeTA global meeting in Geneva in 2014. Study participants consisted of members of MeTA, which included representatives from government, the private sector and civil society. In-depth semi-structured face-to-face interviews were conducted to identify perceptions around the barriers to civil society engagement in the multi-stakeholder process. Interviews were guided by a conceptual framework exploring the three main themes of the political environment, relative stakeholder strength and agenda setting/gatekeepers. Interviews were structured to enable additional themes to emerge and be explored. Fifteen interviews were conducted. The interviews were audio recorded, transcribed verbatim and analysed using a general inductive approach. All interviewees provided written informed consent.

**Results:**

Findings were captured within three main overarching themes: the political environment, relative stakeholder strength and agenda setting/gatekeepers, with the opportunity for additional themes to emerge in the interviewing process. The study conformed these three themes were important in the engagement process. Participants reported that civil society engagement is particularly limited by those who set the agenda. It was largely seen that the political environment was the significant factor that enabled or disabled all others. The findings counter the argument that CSO barriers to engagement are predominantly due to capacity issues.

**Conclusions:**

This study enriches previous findings by providing insights into civil society participation in multi-stakeholder dialogue, specifically the MeTA initiative. The development of more rigorous and systematic accountability mechanisms in order to maintain the legitimacy of decision-making processes and establish more equal power relations would significantly benefit the engagement of civil society organisations. The results inform practical recommendations for MeTA and future multi-stakeholder programmes tasked with improving policy on the access, availability and affordability of medicines.

## Background

Globally, one in three people lacks access to essential medicines [1]. Moreover, when access does exist, medicines can be too expensive, counterfeit or sub-standard, wrongly prescribed or out of stock in the nearest health centre. The pharmaceutical market and medicine supply chains are at the root of these problems; poor information on price and quality, promotion of medicines, distorted competition, corrupt practices and irrational use of medicines [2]. Understanding information about the medical supply chain within health systems is essential to identify how problems should be tackled and by whom. The Medicines Transparency Alliance (MeTA) was established in 2008 as a response to this. MeTA is premised on the idea that making information about medicine supply chains available for analysis by major stakeholder groups will lead to an improved understanding of the problems. This in turn will foster a greater incentive to pioneer change, greater responsibility and accountability upon those needed to instigate change and will lead to increased access to medicines for the most vulnerable sectors of society. In order to achieve its overall goal, MeTA pursued three key objectives; to better inform pharmaceutical policies, to improve practice and to enable multi-stakeholder participation [3]. MeTA sought to improve the access, availability and affordability of medicines for seven countries where access is currently limited (Ghana, Jordan, Kyrgyzstan, Peru, the Philippines, Uganda and Zambia).

At the country level MeTA was developed in response to respective political and social contexts. Whilst being a global alliance, countries were encouraged to shape their structures, priorities and work programmes within existing frameworks to ensure the project would be country led and as sustainable as possible. Each of the pilot countries has organised a Multi-stakeholder Forum, a Council and a Secretariat. One of the most fundamental elements of MeTA in each pilot country was the creation of the national MeTA Councils as multi-stakeholder groups. It was vital that governments, the private sector and civil society organisations (CSOs) were actively engaged as stakeholders in each MeTA council. This created an ‘issue network’; “*a relatively open network of antagonistic actors”*, with a mutual aim, who have expert knowledge and build relationships through information exchange but may subsequently have very different policy ideals [4–8].

The combination of transparency and multi-stakeholder groups may have been used before [9], but applying this model to the area of medicines and health policy was a unique strategy. Indeed, transparency around medicines policy is not a new concept, MeTA’s aim was to creating a multi stakeholder alliance, to generate evidence and translate evidence into policy and practice [10]. Crucially, for CSOs participating in MeTA they were being invited to engage in an area where they had previously found themselves on the margins. Indeed, the involvement of CSOs in policy decision making in general is often limited [10]. The CSOs participating in MeTA consist of community, patient, health, consumer, good governance and transparency groups, media, and faith-based organisations - a network of stakeholders acting in the space between individuals, the state and the private sector. It is, generally accepted that an engaged civil society is central to true democratic legitimacy and promoting accountability [11–14]. Civil Society Organisations (CSOs) have a long history of involvement on health and access to essential medicines, consumer protection and promotion of transparency, including many national as well as international groups. In-country CSOs are focused on health in different ways – as service providers, advocates for rights, or providers of care and support for people with specific health problems. The inclusion of CSOs as one of the three stakeholder groupings in the MeTA pilot was therefore entirely appropriate. However, in a recent MeTA annual report, despite highlighting the considerable advances MeTA has made since the initial pilot phase, reports of obstacles preventing full engagement between CSOs and other stakeholders, were described [15].

To date, little research has been done to explore the full range of factors that cause variation in the ability of CSOs to engage meaningfully in policy development or multi-stakeholder platforms. In the literature, causes of the unequal engagement of civil society are positioned as largely stemming from within civil society as opposed to external factors. Indeed, the most cited factor in determining civil society engagement is the availability of technical capacity [16, 17]. However, for MeTA the barriers to CSO engagement were not perceived as issues arising primarily from poor technical capacity. The issues appeared to be more driven by the domestic, social and political context. Few scholars have tackled the question of what accounts for the variation in civil society engagement beyond capacity issues. Explanations are likely to be insufficient if they do not engage with the reality of the multi-stakeholder process and the dynamics which push and pull decisions and issues both inside and outside of CSOs in different directions. This ‘push and pull’ can be conceptualised as issue emergence and the twin steps of the construction and acceptance of problems as issues. The construction of issues, which consists of definition and framing of a problem, is a key part of issue networks and are logically prior to decisions on issues and solutions championed by networks. This process demonstrates that a stakeholder is responsible for a particular issue and issue acceptance occurs when the issue is championed by at least one major player in the wider network [18, 19]. However, where some actors have more power over decision-making than others, it is likely that CSO perspectives fade into the background if they contradict the policy plans of these gatekeepers. Equally, where the political environment does not welcome the inclusion of civil society opinions (e.g. where engagement is sporadic or where government officials have their own agendas) the likelihood of issue acceptance is threatened. In the case of MeTA, issue acceptance occurs at the internal level of MeTA and at the level of MeTA-government relations.

Despite the limited literature, a number of questions are raised which recognise the influence of factors external to CSOs, such as a highly political policy environment, fear of political opposition and government suspicion [20–22]. Indeed, this resonates with views from a recent CSO meeting, where, *“relative stakeholder strength, local cultural issues (*e.g. *history of democratic process) and agenda setting/threats*” were seen as the key barriers to CSO engagement [15]. In order to explore this issue in more depth this study interviewed MeTA members about their knowledge and experiences of engaging in policy development and multi-stakeholder platforms. Exploring the barriers to CSO participation in MeTA will not only aid in understanding how the policy dialogue can be improved around medicines policy but it will also contribute to the wider discourse on the role of CSOs in multi-stakeholder engagement [20].

## Methods

### Key informants

This study adopted a qualitative approach and was undertaken at the MeTA Global Meeting in November 2014 in Geneva, Switzerland. A list of attendees at the MeTA Global Meeting was obtained prior to the meeting. Participants for the study were sampled purposively based on the following criteria: interviewees must be part of the MeTA alliance (representatives of one of the three stakeholder groups from the participating countries of the alliance), must regularly attend meetings and must speak English or have a translator available. Based on this selection criterion, fifteen participants were recruited for the study and were sent information regarding the study. This included a participant information sheet, which provided an overview of the research study and processes and a research consent form that participants could complete to indicate their interest in participating. This information was also passed on by the researcher conducting the interviews directly prior to the interview itself. Participants were advised that they could withdraw from the study at any time.

### Interviews

A series of face-to-face, semi-structured interviews was undertaken. To ensure consistency, interviews were conducted by the same interviewer. Questions were developed following a review of the relevant literature [1–19] and by identifying suggested key barriers to civil society engagement in the 2014 MeTA annual review. These questions informed a conceptual framework developed to ascertain what actors perceived as barriers to civil society engagement. The conceptual framework focused questions on three main themes: (1) local political environment (2) power imbalances and (3) agenda setting/gatekeepers. Additional questions were used to obtain information about civil society representative’s experiences of multi-stakeholder engagement and their perception of the ways in which they were encouraged or discouraged to voice their opinions. In all interviews, questions were asked about the power relationships between stakeholders within and outside of MeTA and factors which facilitated or inhibited civil society influence on policy change. Questions were asked in English (using a translator where necessary) and were open-ended in order to allow discussions to be led by interviewees as opposed to the interviewer. Demographic information, including sector and country of employment were recorded at the time of the interview. Interviews took place at a quiet location close by to the Global Meeting. Fifteen interviews were conducted, at which stage data saturation was reached. Most interviews were around 50 min in duration (range: 20–50 min). Face to face semi-structured interviews were chosen over qualitative methods due the opportunity to take advantage of social ques and allow for additional themes to emerge. Synchronous communication also gives the advantage of spontaneous answers and direct reaction on answers. All participants were interviewed in the same location to provide a private space for people to partake honestly to enhance the validity of the study.

### Data analysis

After each interview, the interviewer made field notes and this information was used to describe how each interview was conducted and to note issues or comments not sufficiently captured by audio recordings. This information aided interpretation of data. All interviews were audiotaped with the consent of participants. Interviews were transcribed verbatim and the transcripts were checked against the audio recordings. The full transcripts used in the subsequent analysis process. To ensure anonymity, names, place of work, or any other identifying information was removed, and a unique code number was assigned to each transcript. Analysis of data was undertaken by the research team via a staged process to ensure reliability. In the first instance, transcripts were read after each interview to inform further interview questions in order to enable continuous comparison of new and previous interview content. The data were analysed using the general inductive approach (GIA) [21] using the thematic framework as a guide. GIA is a thematic analysis approach with both deductive and inductive features. In GIA, while the general (or overarching) themes are derived from the research objectives (deductive feature), more specific categories/themes arise from the data (inductive feature) [21]. NVivo 10 software (QSR International) was used to support the analysis process. Identification and interpretation of quotes was carried out by two separate members of the research team.

## Results

Of the 15 individuals who participated, four were MeTA country co-ordinators (with civil society backgrounds), two were representatives of the private sector and the remaining nine MeTA members were representatives from civil society. Participants selected represented the following countries; Jordan, Peru, Kyrgyzstan, Zambia, Uganda, Philippines, Ghana. Key findings from the research are presented below.

### The political environment as a barrier to CSO engagement

Despite advances in political freedoms, some MeTA groups operate in political contexts which constrain civil society work and engagement in policy processes. The majority of respondents spoke of the importance of the wider political context to civil society’s ease or difficulty engaging policy. A history of civil society engagement with government in a democratic setting was seen as a clear advantage by all respondents, whereas a lack of this was thought to correspond with difficulties in civil society participation:

*The lack or presence of political will has the power to disable or enable our [civil society’s] ability to act and political will has been seriously lacking in Peru…there is no real history of dialogue between our government and civil society and the government do not want to listen to different perspectives.* [P3]

Regular consultations between civil society and the state were seen as a significant advantage to engagement to civil society representatives in all countries. However, in some countries, consultations with the ministry of health were reported to largely take the form of the provision of evidence for policy change as opposed to providing insight on policy issues. Some actors expressed doubts as to what meaningful action could arise from these consultations. Similarly, the ‘good will’ demonstrated by allowing civil society to participate in consultative stages of the policy process was reported to not translate into further policy action, leaving civil society representatives feeling marginalized:

*Sometimes, it feels like our participation inside and outside of MeTA is just tokenism…we talk and talk and talk but whether anything materializes…well, let’s see.* [P2]

A civil society representative from Peru explained that, while it was originally intended for civil society to provide the government with opinions and recommendations, civil society consultations have been blocked by ministers:

“*…so in the access to essential medicines programme called* Farmacise*, civil society are supposed to provide the government with opinions…recommendations. But the government has blocked that. They don’t want to listen to them specifically in that area. It has been closed for civil society participation so far. That is the big concern in Peru right now.”* [P1]

Unpredictable government engagement with MeTA was also highlighted as a barrier to civil society engagement, as securing government representatives’ presence and input was seen as integral to getting MeTA perspectives represented in policy:

*In Ghana, we had a challenge with the secretariat. That’s finished now. There was a secretariat that was not performing. There today, not there tomorrow. The new one is better, he gives time*. [P9]

In some cases, government officials perceived the presence or voices of CSOs as diminishing or undermining the authority of the state. Perceived former civil society ‘interference’ in policy was also seen as a source of tension by a civil society representative from Ghana, who believed that this had led to government officials being less receptive to civil society opinions. In Kyrgyzstan, civil society has an excellent relationship with the ministry of health but there are tensions between civil society and the Department on Drug Provision and Medical Equipment in the Kyrgyz Republic (DRA). DRA officials were described as a strong barrier to civil society’s ability to influence policy, insofar as it was suggested that they had resorted to defamation of MeTA online. It was suggested that previous civil society recommendations to the ministry of health may be behind this. Tensions with the ministry of health were highlighted in Jordan, where civil society representatives described facing strong pushback and exclusion, most recently concerning negotiations to drive down the price of a Multiple Sclerosis treatment by 50%. According to one civil society representative, sometimes giving civil society a hearing in consultations was used as a way to silence them and prevent civil society action:

*The fight is mainly with the Ministry of Health. The ministry make many promises of policy and price changes to CSOs*… *some believe that the minister of health is stalling and creating delays, cancelling meetings last minute and making promises which are not delivered*…*they don’t just say no as if they did we could bypass them and go directly to the royal court.* [P2]

### Power imbalances

Power imbalances outside of MeTA were described as greatly influencing which actors had a say in policy decisions by the majority of respondents. Government officials were reported to have the strongest voice in Uganda. In Ghana, the government were reported to have the most power inside of MeTA, with this imbalance exacerbated by something of a representational monopoly within MeTA. Until recently, there was only one civil society representative inside MeTA, remedied only after a visit from international members:

*Thankfully, during a visit from international members from the WHO and HAI it was agreed that one person was not sufficient to represent all of the civil society members of MeTA…that proposal sparked resistance from the leadership but the number of representatives was increased to two.* [P5]

In Kyrgyzstan the main perceived barrier to civil society engagement was the DRA’s unchecked power; generally, the DRA was thought to have more influence than the ministry of health on policy and were reported to marginalise the private sector:

*[T]hey [the DRA] can do whatever they want and no one can stop them; if they hate a group or business they can put a lot of pressure on them*… *They cannot talk about this openly or they will be punished: they just try to use civil society because the regulatory agency have such huge power and can put huge pressure on them*. [P13]

Conversely, in Peru, Zambia, Ghana and Jordan, respondents voiced the opinion that the private sector not only had a stronger voice than civil society outside of MeTA but was also more powerful than governments. This manifested in a number of ways, for example by having the power to appoint or remove government officials or influence policy by leveraging their profits and influence: One respondent also highlighted the unchecked power of procurement officers, who had the power to procure certain medicines and supplies, influencing buying patterns of medicines procurement for their own profit.

### Gatekeepers and agenda setting

Where civil society positions did not correlate with the government’s own agenda, ministers’ own policy agendas were often identified as a strong factor in inhibiting or enabling civil society participation outside of MeTA. In most countries, the strength of government depended on the relative importance of certain issues to ministers.

The active involvement of government officials inside the MeTA process was widely seen as greatly beneficial to raise the profile of issues with governments. However, although having members of government in powerful positions within MeTA was reported to increase engagement, many respondents expressed ambivalence. Where government engagement within MeTA was necessary to influence policy, sporadic engagement from government officials represented a hurdle to civil society engagement, as in Ghana and Uganda:

*The strength of the ministry of health in Uganda barrier to things getting done because if they are not at the table you have to find a way of getting them to the table; if they are not in meetings…for example, this meeting, we feel that it makes our jobs harder.* [P11]

Concerns about having government representatives in powerful positions within MeTA were raised by a participant, who emphasised possible conflicts of interest and difficulties in ensuring that some stakeholders do not have unchecked power:

*For example, there might be changes in policy that work towards the interests of the government or the private sector. If the government is the leading member of the MeTA council, how can this be balanced? Who will balance it? How can you be critical if you need them to sign off on every policy change? So I think that would be a difficult position. Where will people go if they have problems with the government? I don’t know what the dynamics would be like [sic] there, if that would be the system.* [P2]

In some cases the disproportionate influence of government officials outside of MeTA was reported as influencing which conversations were conducted within MeTA. Some respondents referred to powerful individuals within MeTA having their own policy agendas and using their position to influence which positions MeTA could take.

*It makes a difference in what we can say…because they are the ones who are listened to, they are the ones who are consulted by government….If there are agitations of any kind the president of the Ghana association is the last person who needs to be consulted. So he keeps quiet, you bring the draft to him…he has the final decision…if he does not like a point he just sends it back.* [P5]

Some interviewees emphasised being non-confrontational and avoiding making demands in order to be heard. Where power imbalances were reported to be minimal, this simply took the form of all stakeholders “agreeing to disagree” [P2]. In Kyrgyzstan it was noted that, due to tensions with the DRA, civil society members are careful to mediate their interactions with the DRA. Conversely, respondents in countries where engagement was low reported taking care when approaching government officials, as they were thought to have a large amount of power in the policy process within and outside of MeTA. Their presence not only influenced the agenda but also how debates were conducted. For example, in Zambia gaps in services can be highlighted by civil society, but not expressed as being the result of government shortcomings:

*Our approach is not confrontational. When we speak with government…if all we do is point fingers and accuse them of things, nothing happens. We have to engage in more…civil ways.* [P8]

### Capacity

Interview responses revealed key variations and similarities in the factors viewed as facilitating and inhibiting civil society participation. Contrary to the existing literature, capacity was not seen as a barrier to engagement, it was however frequently referenced and therefore an overview of respondents’ views of capacity is also presented.

While room for further civil skills strengthening with regards to policy was recognised every interviewee stated that technical capacity to engage in the area of medicines policy as part of MeTA was strong: In all countries, capacity was thought to be strengthened through the inclusion of members with previous technical or policy experience due to the skills and recognition they brought to MeTA:

*We have the capacity. We have members who are trained in health and have had advocacy training, Master’s degrees, we have retired medical and pharmaceutical officers. All of these things help us to be taken seriously.* [P5]

Representatives from all countries noted that civil society engagement had been strengthened through capacity-building activities run by MeTA. A civil society respondent from Uganda stated that whereas previously the Ugandan government had accused civil society of “*just being noisy*”, now the government is aware that civil society within MeTA “*use evidence to get the change that we want”*. A similar view was expressed by a civil society representative from Jordan, where civil society did not previously possess the technical skills for evidence-based advocacy. Outside of MeTA, views of civil society capacity were not always positive. For example, a respondent from Zambia expressed a belief that members of professional associations did not necessarily believe that civil society had the capacity to engage in policy. A civil society respondent reported that heads of hospitals also had doubts about civil society capacity and perceived civil society as interfering with their day to day functioning:

*Heads of hospitals, they get a certain level of responsibility and autonomy with their budgets…they have a lot of freedom in how they spend it. They don’t want anyone messing up their good life, so they say, “Who are these people? What do* they *know about medicines”?* [P10]

A number of respondents expressed the opinion that, while capacity is an important factor in engaging with policy, it was not the strongest predictor of civil society success in the multi-stakeholder process; barriers were most often perceived in getting powerful stakeholders to engage once a certain level of capacity had been achieved:

*It doesn't matter how high our capacity is. At the end of the day, if we cannot engage with government in a way they appreciate it just doesn’t work.* [P15]

## Discussion

This research explored the opinions and perceptions of civil society organisations in the MeTA multi-stockholder dialogue process. Furthermore, the study used MeTA as a case study to confirm the factors which limit civil society engagement in multi-stakeholder dialogue. These findings advance our understanding of multi-stakeholder dialogue within the medicines policy arena and provide further insights into how we can effectively foster transparent and open information sharing to create improved policy surrounding access to medicines. The study used in-depth interviews to explore three key themes relevant to the variations seen in CSO engagement within MeTA; (1) local political environment (2) power imbalances and (3) agenda setting/gatekeepers. The study also allowed for the emergence of new themes in order to gain a deeper understanding and identify where improvements might be made.

Prior research has shown that a lack of civil society capacity can seriously impact civil society's ability to engage with policy [16]. However, capacity-building strengthening activities carried out by MeTA did not have any effect on engagement [15]. Despite stating that they had good technical capacity, many civil society representatives did not perceive themselves as well-positioned to influence policy. Capacity was not identified as a current barrier to civil society engagement by any respondent in this study. This was the case not only in countries where civil society engagement is strong, but also in those countries where engagement was reported to be comparatively weak in a recent MeTA annual review [15].

Of the three main themes explored, political environment was deemed especially important, and the ultimate deciding factor for effective collaboration. In countries where government’s commitment to policy change in the area of access to medicines is strong, civil society find dialogue and collaboration more consistent and productive than CSOs do in those countries where commitment and multi-stakeholder engagement is weak. In addition, where governments offer an open consultation process to civil society, engagement is higher than in political systems in which governments regularly block policy consultations or changes proposed by civil society.

Unequal power dynamics and relative stakeholder strength outside of MeTA were seen to hamper civil society engagement on a number of levels. Firstly, where the private sector has more influence than civil society due to a higher degree of political currency in consultations or the ability to leverage monetary resources, civil society are disadvantaged. The powerful position of other stakeholders such as procurement officers, hospital heads and government officials were also cited as an issue. Ultimately, wherever political will was lacking or the private sector had a disproportionate influence on the direction of policy, civil society are disadvantaged. Power imbalances strongly influence the extent of ownership felt by actors over issues or solutions which emerge within MeTA. Actors have varying levels of power within MeTA and power imbalances were evident, particularly where civil society were underrepresented or only involved in consultative processes. Issue networks themselves do not have policy goals; these arise from actors within the network and some actors have more power than others in the issue formation process. Furthermore, if issue definition involves demonstrating that a party is responsible for a certain situation and proposing credible solutions, a culture of having to be careful in ‘blaming’ government officials for previous mistakes may stand in the way of truly participatory engagement for civil society [18].

The issue of gatekeepers and agenda setting as a barrier to policy engagement was most commonly raised regarding government officials and the position they hold within MeTA. Indeed, it is suggested that in countries with a less hospitable political environment to civil society, issue adoption often occurs when the issue is championed by one major player in the broader network [17]. Gatekeepers are seated in powerful positions within MeTA due to their power to lend credibility, sources or pathways to a raised ‘issue’. These benefits were widely discussed and seen as integral to MeTA’s policy influencing activities. However, gatekeepers can also block the entry of policy issues into the policy process; gatekeepers have “*powerful demonstration effects, signalling that certain causes are important*” [19]. If gatekeepers disagree with civil society positions, this represents a significant disadvantage for civil society, insofar as their views may be silenced [23]. Here, it is likely that the policy suggestions most likely to reach policy makers are those most well-aligned to the positions already held by the policy maker or the wider government department. Conversely, the position of civil society is at its strongest within MeTA when the process of issue formation was closest to the principle of collaborative governance [24, 25]. That is, where stakeholders representing different interests make policy decisions or recommendations to a final decision-maker who does not substantially change consensus recommendations.

There is a sophisticated academic debate on the ‘democratic deficit’ in global policy-making and the approaches necessary to make public policy-making more accountable [26]. Despite this, issues of accountability were rarely, if ever, explicitly discussed during the interviews. It is argued that multi-sectoral networks should be embedded in a pluralistic system of accountability, making use of a combination of accountability, as outlined by Benner and colleagues [27]. For example, they may employ measures of “internal accountability” (oversight committees) or “reputational accountability” (naming and shaming) [27]. It is likely that more rigorous and systematic accountability mechanisms are needed within MeTA in order to maintain the legitimacy of decision-making processes. Overall, further research into the power structure of local MeTA councils and meetings would be useful in order to identify the conditions under which stakeholders act collaboratively. This study suggests that, particularly where government officials are serving as chairs, clear guidelines to government engagement within MeTA should be formulated in order to ensure that proper checks and balances are put in place. This would represent a step towards ensuring that no one actor has the power to sign off on policy changes within MeTA. It is unclear whether any government officials involved with MeTA have been systematically trained in civil society relations but, although actors would still be confined by the overarching political environment, such training may prove useful in ensuring that there is enough space for diversity of opinion and debate both within and outside of MeTA. Furthermore, clearly defined goals and specific expected outputs should be designed by each MeTA country group in order to focus and motivate MeTA members and ensure accountability. In this way, actors may be more open to the strategies of other sectors if these strategies appear useful for achieving agreed upon goals. It may be useful to complement this with some frank discussion about perceptions of power relations within and outside of MeTA, the various sources of influence around the table and the strategies needed to address any problematic power differentials. This may involve the use of professional facilitation or introduction of specific measures to “*level the playing field”* through, for example, formalised power-sharing rules, or increased representation of “weaker” stakeholder groups.

The qualitative nature of the study means that this research draws heavily on the personal experiences of civil society members. While this is an advantage insofar as we have gained an in-depth understanding of their perceptions of the multi-stakeholder process, a reliance on personal reflections limits this paper’s reach to some extent by relying on respondents’ ability to recall events or actions. The possibility of participants wanting to shine a favourable light on the work of their own organisations should also be considered, as should wishes not to offend country governments by portraying them as unresponsive. Furthermore, the use of convenience sampling in selection of study participants may lead to bias due to the fact any civil society members attending the conference in Geneva may possibly be more engaged in the policy process than other civil society counterparts.

In reality, actors in a network may come together because of disagreements over the presence of a solution to certain issues. The practice of collaboration has long demonstrated that multi-stakeholder partnerships and relationships are often fraught, with many facing significant setbacks in delivering the resources or outcomes originally stated. Even where preliminary milestones have been achieved, multi-stakeholder groups often still experience collaborative inertia which can have significant effects on outputs [27]. In light of these challenges, MeTA serves as an interesting case study to explore how civil society are encouraged or prevented from voicing opinions and influencing policy in a meaningful way without assuming shared agendas.

## Conclusion

This study enriches the current understanding of civil society engagement in multi-stakeholder dialogue, by providing ginsights into the opinions and perceptions of CSO’s participating in a multi-stakeholder alliance, MeTA, aimed at improving access to medicines policy. The studies confirms that the political environment, relative stakeholder strength and gatekeepers have a significant role in CSO engagement within the MeTA issue network. However, it was largely seen that the political environment was the one factor that enabled or disabled all others. In this case, the findings counter the argument that CSO barriers to engagement are predominantly due to capacity issues. It is evident that there is no one-size-fits-all template for civil society engagement: any interventions in these relationships must be tailored to their specific contexts and goals. However, for MeTA it is likely that the use of more rigorous and systematic accountability mechanisms in order to maintain the legitimacy of decision-making processes and establish more equal power relations would significantly benefit the engagement of CSOs. This study provides a significant starting point for a discussion about potential barriers and solutions for improving transparency, accountability and evidenced-based policy making in improving access to medicines through multi-stakeholder dialogue.

## Abbreviations

CSO: Civil Society Organisation
DRA: Drug Regulatory Authority
MeTA: Medicines Transparency Alliance
NGO: Non-governmental organisation
WHO: World Health Organization
